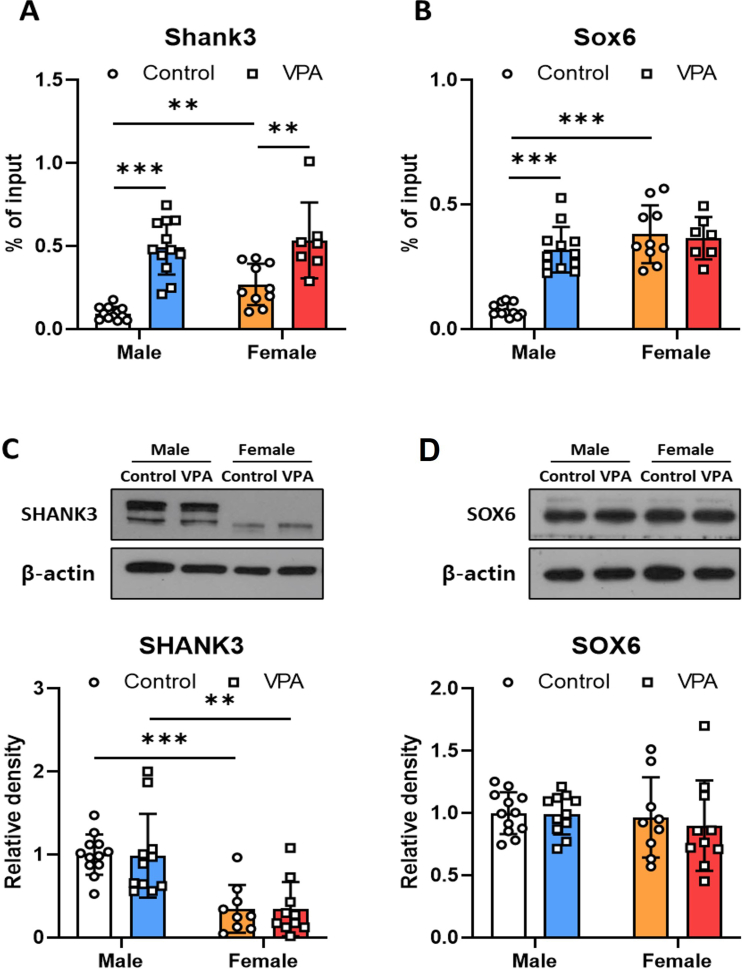# Investigation of long-term epigenetic changes in the Nr3c1 gene by neonatal valproate exposure in juvenile rats

**DOI:** 10.1016/j.gendis.2025.101588

**Published:** 2025-03-08

**Authors:** Eun Hye Jang, Soon Ae Kim

**Affiliations:** Department of Pharmacology, School of Medicine, Eulji University, Daejeon 34824, Republic of Korea

Neurodevelopmental disorders such as autism spectrum disorder (ASD), are complex conditions influenced by both genetic and environmental factors. Epigenetic changes serve as a critical bridge between these factors, potentially mediating the effects of environmental exposures on gene expression patterns crucial for neural development. These modifications, occurring during pregnancy or early postnatal life, could have long-term effects on genes associated with neurodevelopment, increasing the risk of neurodevelopmental disorders. Valproic acid (VPA) is linked to an increased risk of ASD when exposure occurs *in utero*.^1^ VPA-exposed rodent models accurately reflect human ASD conditions, demonstrating changes in social behaviors and glial precursor proliferation that may influence synaptic connectivity, neuroinflammation, and the integrity of the blood–brain barrier, all of which are implicated in ASD pathophysiology.^2^ VPA inhibits histone deacetylase, leading to histone acetylation (*e.g.*, H3K27ac) and affecting the epigenetic regulation of genes involved in neurogenesis and transcription factors such as *Ascl1*^3^ and *Foxo3*.^4^ This study investigates epigenetic mechanisms, including histone modification and DNA methylation, impacting brain development in neonatally VPA-exposed rats.

In this study, male and female Sprague–Dawley rats were used. On postnatal days 2 and 3, neonates received subcutaneous injections of 300 mg/kg VPA or saline twice daily, with a single injection on day 4. Ethics approval for the animal experiments was obtained from the Institutional Animal Care and Use Committee (IACUC) of Eulji University (EUIACUC 20-05). At ages 4 or 8 weeks, rats were euthanized, and cortices and hippocampi were extracted. Chromatin was isolated from these tissues, and preliminary chromatin immunoprecipitation (ChIP) sequencing was conducted using an acetyl-H3K27 antibody with a pooled cortex sample per group in the cortices of 4-week-old male rats. ChIP-sequencing data were then analyzed with MAnorm to identify differential binding across the groups. Enhanced H3K27ac at distal intergenic regions might suggest increased activation of enhancer elements in response to VPA (Fig. S1). To ensure statistical rigor, we applied a false discovery rate correction and selected regions with a −log_10_(*p*-value) ≥ 5 as significant. ChIP sequencing revealed significant enrichment in H3K27 acetylation at 326 genes (Table S1). Among these significant regions, genes known as autism candidate genes were prioritized, with validation through the SFARI Gene database (https://gene.sfari.org/). This targeted selection enabled us to focus on high-confidence loci relevant to ASD pathophysiology. Gene Ontology (GO) analysis identified significant enrichment in biological processes such as gliogenesis (false discovery rate *p* < 0.05, Table S2). Candidate genes including *Mapk1*, *Shank3*, *Sox6*, *Pax6*, *Foxp1*, *Ubr4*, and *Nr3c1* were selected for further analysis. Quantitative PCR (qPCR) for mRNA expression and ChIP-qPCR were used to assess changes in gene expression of these candidate genes (Tables S3 and 4). Using ChIP-qPCR, significant H3K27ac enrichment at the *Shank3* promoter was observed in both male and female VPA-exposed neonatal rats. Increased *Sox6* promoter enrichment was noted only in males. However, only sex differences in SHANK3 protein levels were observed in the Western blot analysis and two-way ANOVA analysis results confirmed significant sex effects on SHANK3 but not SOX6 protein levels (Fig. S2).

Previous studies have demonstrated that neonatal VPA exposure leads to decreased *Nr3c1* mRNA expression in both male and female cortices.^5^ Additionally, Western blot analysis confirmed reduced glucocorticoid receptor (NR3C1) protein levels specifically in the cortices of neonatal VPA-exposed male rats. In male rats neonatally exposed to VPA, a discernible reduction in H3K27ac-associated DNA fragments in the *Nr3c1* promoter region was observed compared with the control group. This alteration was male-specific, with no significant changes detected in the cortex or hippocampus of female rats (Fig. 1A: male, *t* (11.724) = 5.693, *p* < 0.001; Fig. 1B: male, Mann–Whitney U, *p* = 0.038). Two-way ANOVA showed significant effects of sex (*F* = 33.867, *p* < 0.001) and VPA treatment (*F* = 24.128, *p* < 0.001) on the cortex tissue samples, with significant interaction between these factors (*F* = 19.418, *p* < 0.001). This suggests a synergistic effect where the combination of sex and VPA treatment influenced gene expression more profoundly than either factor alone.

**Figure 1:**
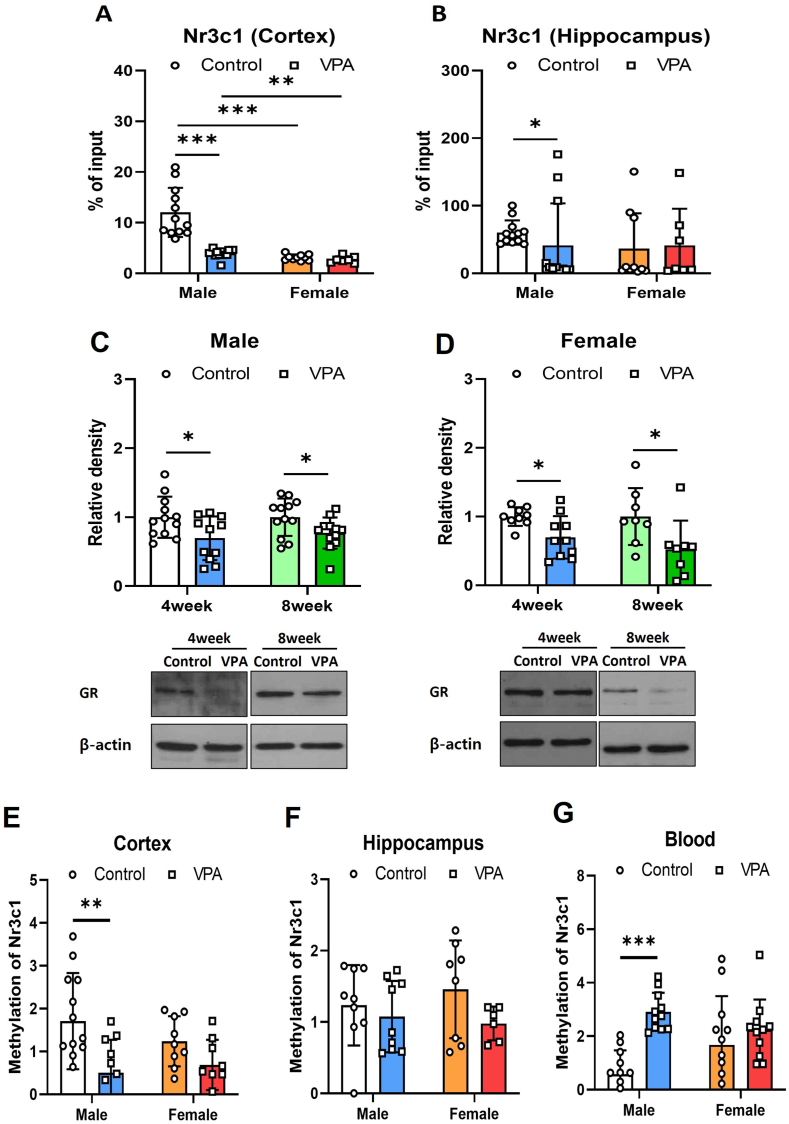
Epigenetic features of the *Nr3c1* promoter region in tissue- and sex-specific contexts of a neonatal VPA exposed animal model. **(A, B)** H3K27 acetylation of the promoter region of *Nr3c1* was assessed by ChIP-qPCR in the cortex (A) and hippocampus (B) of the VPA neonatally exposed 4-week-old rats (male control, *n* = 12; male VPA, *n* = 12; female control, *n* = 10; female VPA, *n* = 7). **(C, D)** NR3C1 protein levels in male (C) and female (D) rats were measured by western blotting in VPA neonatally exposed 4- and 8-week-old rat hippocampus (C: 4-week-old male control, *n* = 12; 4-week-old male VPA, *n* = 11; 8-week-old male control, *n* = 12; 8-week-old male; VPA *n* = 12; D: 4-week-old female control, *n* = 9, 4-week-old female VPA, *n* = 10; 8-week-old female control, *n* = 8; 8-week-old female VPA, *n* = 8). **(E**–**G)** Methylation of the promoter region of *Nr3c1* was assessed by MSRE-based qPCR in the VPA neonatally exposed 4-week-old rat cortex (E), hippocampus (F), and blood (G). (E: male controls, *n* = 13; male VPA, *n* = 11; female control, *n* = 9; female VPA, *n* = 9; F: male control, *n* = 9; male VPA, *n* = 9; female control, *n* = 8; female VPA, *n* = 6; G: male control, *n* = 12; male VPA, *n* = 11; female control, *n* = 12; female VPA, *n* = 11). The values represent mean ± standard deviation. The significance level between groups is indicated by ∗*p* < 0.05, ∗∗*p* < 0.01, and ∗∗∗*p* < 0.001. ChIP, chromatin immunoprecipitation; qPCR, quantitative PCR; MSRE, methylation-sensitive restriction enzyme; VPA, valproic acid; NR3C1, nuclear receptor subfamily 3 group C member 1.

Western blotting was performed to investigate the impact of neonatal VPA exposure on the protein levels of the glucocorticoid receptor in both male and female hippocampi. Glucocorticoid receptor protein levels were reduced in 4-week-old rats after VPA exposure (Fig. 1C; male, *t* (21) = 2.388, *p* = 0.026; female, *t* (22) = 2.258, *p* = 0.034) and persisted until 8 weeks of age (Fig. 1D; male, *t* (12.758) = 2.812, *p* = 0.034; female, Mann–Whitney U *p* = 0.027). These results underscore the necessity of examining multiple brain regions to fully elucidate the epigenetic and gene expression changes induced by environmental changes like neonatal VPA exposure.

The methylation level of the *Nr3c1* promoter in the cortex was reduced in 4-week-old male rats exposed to VPA, but no changes were observed in female rats (Fig. 1E; *t* (22) = 3.010, *p* = 0.006). Two-way ANOVA showed that there was no significant effect of sex (*F* = 0.313, *p* = 0.579) or interaction between sex and VPA treatment (*F* = 1.577, *p* = 0.217) on the methylation status in the cortex tissue. However, the effect of VPA treatment was significant (*F* = 11.325, *p* = 0.002), indicating that VPA treatment alone significantly altered the DNA methylation patterns in the cortex, independent of sex (Fig. 1E). Figure 1F focuses on the brain hippocampus and revealed that neither sex (*F* = 0.102, *p* = 0.752), VPA treatment (*F* = 2.722, *p* = 0.110), nor their interaction (*F* = 0.682, *p* = 0.416) significantly affected DNA methylation levels. This suggests that DNA methylation in the hippocampus is not significantly influenced by neonatal VPA exposure or sex, highlighting a possible regional specificity in the epigenetic response to environmental factors. In blood samples, sex (*F* = 0.337, *p* = 0.564) did not show significant effects on DNA methylation levels; however, VPA treatment (*F* = 15.963, *p* < 0.001) and the interaction between sex and VPA treatment (*F* = 6.041, *p* = 0.018) were significant in the two-way ANOVA analysis results. Notably, contrary to findings in brain tissue, DNA methylation levels at the *Nr3c1* promoter in the blood of 4-week-old males exposed to VPA were significantly increased (Fig. 1G, *t* (21) = −6.902, *p* < 0.001), indicating that VPA neonatal exposure significantly influences DNA methylation in blood, inconsistent with that in the brain tissues, such as cortex or hippocampus. This highlights the complex nature of tissue-specific responses in the DNA methylation of the *Nr3c1* promoter between the blood and brain in the VPA neonatally exposed 4-week-old male rats. These differences could reflect distinct cellular compositions, epigenetic landscapes, and environmental exposures between peripheral and central nervous system tissues.

These epigenetic changes in H3K27ac and DNA methylation may influence gene expression, especially *Nr3c1*, in neurodevelopmental processes such as synaptic development, stress response, and neuroinflammation, which are implicated in ASD. Tissue-specific methylation differences also suggest potential biomarkers reflecting neurodevelopmental changes, warranting further research into their role in behavioral and cognitive outcomes. We recognize limitations in our analysis, including a focus on promoter regions, assessment of DNA methylation at a single site, and analysis of only H3K27ac modifications, which may not capture the full epigenetic landscape. Additionally, our post hoc power analysis suggested limited statistical power for some comparisons. Future studies should examine epigenetic changes across broader genomic regions and developmental stages, employ larger sample sizes to improve robustness and consider potential confounding factors to enhance interpretive accuracy.

## CRediT authorship contribution statement

**Eun Hye Jang:** Writing – original draft, Visualization, Methodology, Investigation, Formal analysis, Data curation. **Soon Ae Kim:** Writing – review & editing, Writing – original draft, Supervision, Investigation, Funding acquisition, Data curation, Conceptualization.

## Ethics declaration

Ethics approval for the animal experiments was obtained from the Institutional Animal Care and Use Committee (IACUC) of Eulji University (EUIACUC 20-05).

## Funding

This work was supported by Eulji University in 2023 (EJRG-23-18) and a National Research Foundation of Korea (NRF) grant funded by the Korean government MIST (No. 2020R1A2C1009499).

## Conflict of interests

The authors declared no conflict of interests.

## Figures and Tables

**figs1:**
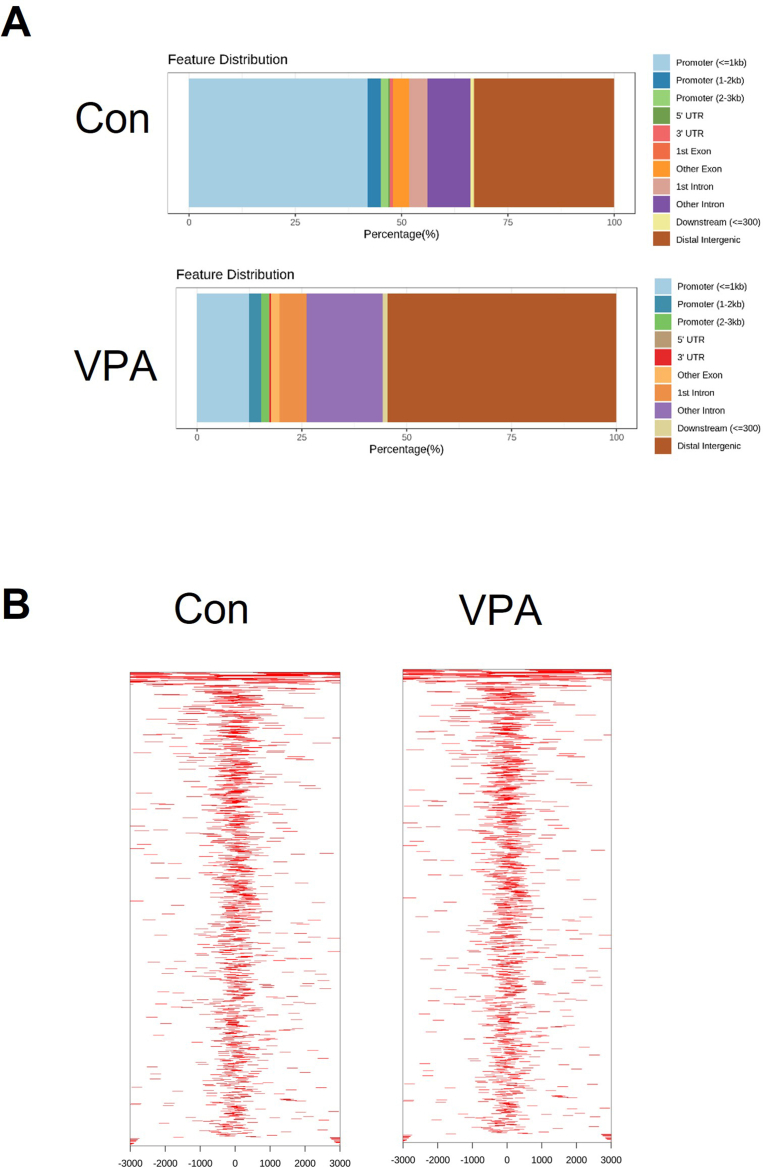


**figs2:**