# Supplementary material for: Investigation of long-term epigenetic changes in the Nr3c1 gene by neonatal valproate exposure in juvenile rats
Source: Genes Dis. 2025 Mar 8;12(6):101588. doi: 10.1016/j.gendis.2025.101588 (PMC12305572; doi:10.1016/j.gendis.2025.101588)
Supplement: Multimedia component 3 [file mmc3.docx]

**Table S3. Primer sequence list**

| **Primers for RT-qPCR** | | |
| --- | --- | --- |
| Gene name | Forward sequence (5'→3' | Reverse sequence (5'→3') |
| *Gapdh* | AGTGCCAGCCTCGTCTCATA | AGAGAAGGCAGCCCTGGTAA |
| *Foxp1* | CAGTCCAGGCAGATCCCCTA | TGTGGTTGGCTGTTGTCACT |
| *Ubr4* | GCACCATCAACTGGCAGAAG | CCGCAAAGAGCACAGGAAAG |
| *Shank3* | TGAGGCCAGACATTGCAGAC | ACAATGAGCTGATCTCGGCA |
| *Mapk1* | CCAGGATACAGATCTTAAATTGGTC | GGAACGGCTCAAAGGAGTCA |
| *Sox6* | CCAGAGGACGCAGAAGGAAG | CCTCGGGCATCCCTGTAGA |
| *Pax6* | TCGGATGAGGCTCAAATGCG | TTGGCTGCTAGTCTTTCCCG |
| **Primers for ChIP-qPCR** | | |
| Gene name | Forward sequence (5'→3' | Reverse sequence (5'→3') |
| *Shank3* | CAAGCACAGGTTCATTGGGC | AGTTGGGGTAAAGTCTAGCTGC |
| *Sox6* | GTTAGATGGACGTCACTGCAC | GGAATTCCAGCCAGAGATAAAAGG |
| *Nr3c1* | TGGTAGTTTTGACTAGACCTGGTT | TCTCACCCAGTGTGGACATC |
| **Primers for MSRE-based qPCR** | | |
| Gene name | Forward sequence (5'→3' | Reverse sequence (5'→3') |
| *Nr3c1* | GTTTCCGTGCCATCCTGTAG | GTGGGTCGGCAGCCAGAGC |
