# Supplementary material for: Investigation of long-term epigenetic changes in the Nr3c1 gene by neonatal valproate exposure in juvenile rats
Source: Genes Dis. 2025 Mar 8;12(6):101588. doi: 10.1016/j.gendis.2025.101588 (PMC12305572; doi:10.1016/j.gendis.2025.101588)
Supplement: Multimedia component 4 [file mmc4.docx]

**Table S4. mRNA expression results of candidate genes based on ChIP-seq data analysis in neonatal VPA exposed 4 weeks old male cortex**

| Gene name | Control | VPA | *p*-value  (vs control) |
| --- | --- | --- | --- |
|  | Mean ± SD | Mean ± SD |  |
| *Foxp1* | 1 ± 0.36 | 1.67 ± 0.43 | < 0.001 |
| *Ubr4* | 1 ± 0.23 | 1.56 ± 0.25 | < 0.001 |
| *Shank3* | 1 ± 0.27 | 1.13 ± 0.27 | 0.045 |
| *Mapk1* | 1 ± 0.37 | 1.89 ± 0.61 | < 0.001 |
| *Sox6* | 1 ± 0.77 | 2.32 ± 0.82 | < 0.001 |
| *Pax6* | 1 ± 0.3 | 1.9 ± 1.06 | 0.019 |
