# Supplementary material for: Investigation of long-term epigenetic changes in the Nr3c1 gene by neonatal valproate exposure in juvenile rats
Source: Genes Dis. 2025 Mar 8;12(6):101588. doi: 10.1016/j.gendis.2025.101588 (PMC12305572; doi:10.1016/j.gendis.2025.101588)
Supplement: Multimedia component 5 [file mmc5.docx]

**Materials and methods**

*Animal model*

Male and female Sprague–Dawley rats, aged seven weeks, were acquired from Samtako (Gyeonggi-do, South Korea). Upon arrival, the animals underwent a one-week acclimatization period where they were housed under a standard 12-hour light-dark cycle at a controlled temperature of 22℃ (± 2℃) and humidity (50% ± 10%). They had unrestricted access to food and water. At eight of age, breeding was initiated by pairing one male with two females. The pregnant females were then systematically assigned to one of two experimental groups. The birth of the neonates was designated as postnatal day zero (PND 0), with litter sizes typically ranging from 7 to 15 pups. On PND 2 and 3, all neonates received subcutaneous injections of either 300 mg/kg of VPA (Sigma-Aldrich, St. Louis, MO, USA) or a saline solution twice daily, and a single injection on PND 4. Each treatment group included at least three pregnant females, and all offspring from the same mother were included in the experiment. We conducted a power analysis using G*Power (version 3.1.9.7), with α set at 0.05 and desired power of 0.80. For a t-test (Means: Wilcoxon-Mann-Whitney test) with an effect size 𝑑=2, the required sample size per group was determined to be 4. Additionally, for an F-test (ANOVA: Fixed effects, special, main effects and interactions) with an effect size 𝑓=1 and numerator degrees of freedom set to 1, the required total sample size was calculated to be 11. At the ages of 4 or 8 weeks, the rats were euthanized using inhalation anesthesia, after which their cortices and hippocampi were extracted. The samples were stored at -80℃ until further experiments. This entire study protocol was sanctioned by the Institutional Animal Care and Use Committee of the Eulji University (EUIACUC 20-05).

*Chromatin immunoprecipitation and sequencing (ChIP-seq)*

Chromatin was extracted from the cortex and hippocampal tissues using a chromatin isolation kit (Abcam, Cambridge, UK) following the manufacturer's protocol to ensure consistent quality and integrity. The chromatin immunoprecipitation (ChIP) assay was conducted using the ChIP kit-One Step protocol (Abcam), which simplifies the procedure by integrating several steps into a streamlined workflow. For immunoprecipitation, we utilized an acetyl-H3K27 antibody (Abcam), a widely used marker of active enhancers, to capture acetylated H3K27 histones specifically. Following ChIP, the extracted DNA was quantified using the Quant-IT PicoGreen dsDNA Assay Kit (Invitrogen, Carlsbad, CA, USA), a highly sensitive fluorescence-based assay designed to accurately measure low-concentration DNA. DNA quality and fragment size distribution were assessed on the Agilent 2100 Bioanalyzer using the High Sensitivity DNA Kit (Agilent Technologies, Santa Clara, CA, USA), ensuring DNA was of sufficient quality for subsequent library preparation.

*Library Preparation and Sequencing*

ChIP-seq libraries were prepared according to the manufacturer’s guidelines using the TruSeq ChIP Sample Preparation Kit (Illumina, Inc., San Diego, CA, USA). This kit is optimized for constructing libraries from ChIP-DNA, enabling efficient adapter ligation and amplification. Library quantification was performed using quantitative polymerase chain reaction (qPCR) following the KAPA Library Quantification kits for Illumina Sequencing platforms (KAPA Biosystems), as per the qPCR Quantification Protocol Guide. To further confirm library quality and concentration, we used the TapeStation D1000 ScreenTape (Agilent Technologies), which allows for precise quality control of DNA fragment distributions. Indexed libraries were sequenced on the Illumina NovaSeq™ platform at Macrogen Incorporated. Each sample was sequenced to a depth of 3 Gb, generating approximately 15 million paired-end reads per sample. This depth ensured robust coverage and sensitivity in detecting enrichment peaks across the genome.

*Sequencing Consistency and Quality Control*

To maintain data reliability, all samples were sequenced under consistent conditions with identical sequencing parameters, including read depth and quality. Comprehensive quality control was performed on each sample prior to analysis, using standard metrics such as read quality scores (Q30), base composition, and GC content, ensuring high-quality sequencing data. Any samples not meeting these quality criteria were re-sequenced to maintain consistency across the dataset. For transparency, the raw sequencing data will be made available upon request to facilitate further validation and analysis (Supplementary Data).

*Data processing*

Sequencing quality was initially assessed with FastQC (version 0.11.7), and adapter sequences were removed using Trimmomatic (version 0.38) with a sliding window quality threshold of 15 and a minimum read length of 36 bp. The cleaned reads were aligned to the rat genome (rn6) with Bowtie (version 1.1.2), allowing up to two mismatches per seed and retaining uniquely mapped reads. PCR duplicates were removed using the MarkDuplicates module in Picard (version 1.118). Peaks were identified MACS2 (version 2.1.1), with a significance threshold set to an FDR of 5%, to determine enriched regions with high confidence. The ChIPseeker (version 1.18.0), a bioconductor package in R (version 3.5.1) used to facilitate batch annotation of enriched peaks, was used for annotating peaks and identifying genomic regions, such as TSS, exon, intron, and intergenic regions. MAnorm was used to compare the differences in the peaks between the two groups. Checking for overlaps of the called peaks in each group and statistical testing of quantitative comparisons between the two groups using read coverage for each peak region were performed. To gain insights into the functional significance of the genomic regions enriched with H3K27acetylation marks, we performed Gene Ontology (GO) analysis using the PANTHER (<https://www.pantherdb.org/>).

*ChIP quantitative polymerase chain reaction (ChIP-qPCR)*

Primers were designed based on the promoter region of the candidate gene. The primer sequences are presented in Table S1. SYBR Green Supermix (Bio-Rad, Hercules, CA, USA) and the CFX96TM Real-Time System (Bio-Rad) were used for amplification with primers. The following PCR conditions were used: 95℃ for 10 min, 50 cycles of 95℃ for 15 s, and annealing temperature for 1 min. The percentage of target genes in each sample was calculated by comparing the Ct values of the input and ChIP DNA.

*Real-time quantitative polymerase chain reaction (qPCR) analysis*

Total RNA was extracted from the hippocampus using the RNeasy Mini Kit (Qiagen, Hilden, Germany). Complementary DNA (cDNA) was synthesized using an iScript^TM^ cDNA synthesis kit (Bio-Rad) from 100 ng of total RNA. cDNA was mixed with SYBR Green Super Mix (Bio-Rad) and primers and amplified using the CFX96TM Real-Time System (Bio-Rad). The primer sequences used to amplify the target genes are listed in Table S1. mRNA expression was normalized to that of *Gapdh*. Raw data were analyzed using the 2^-ΔΔCT^ method.

*Western blotting*

Tissue proteins were isolated using RIPA buffer (ATTO, Tokyo, Japan) containing proteinase and phosphatase inhibitors (ATTO). The protein concentration was measured using a BCA assay kit (Thermo Fisher Scientific, Waltham, MA, USA). Proteins (20 µg) were separated on SDS-PAGE gels and transferred onto nitrocellulose membranes (Pall, Port Washington, NY, USA). The membranes were blocked with 5% non-fat milk in a TBST buffer for 1 h at 4℃ and incubated with primary antibody diluted in TBST at 4℃ overnight. The membranes were rinsed with TBST and incubated with horseradish peroxidase-labeled secondary antibodies diluted in TBST for 1 h. Antibodies used are listed in Table S2. After washing with TBST buffer, the membranes were incubated with West Femto Maximum Sensitivity Substrate (Thermo Scientific, Waltham, MA, USA). Protein expression levels were detected by exposure to X-ray film (Agfa, Mortsel, Belgium) and analyzed using ImageJ lab software https://imagej.net/ij/ (NIH, Bethesda, MD, USA)

*Methylation-sensitive restriction enzymes (MSRE)-based qPCR*

Total genomic DNA was extracted from the hippocampus, cortex, and blood using the DNeasy Blood and Tissue Kit (Qiagen, Hilden, Germany). DNA (1 μg) of each sample was diluted in 50 μL rCutsmart buffer^TM^ (Biolabs, Boston, MA, USA) and then divided into three aliquots. Two aliquots were digested at 37°C for 1 h with *Msp*I (Biolabs) or *Hpa*II (Biolabs) and one aliquot was not digested. SYBR Green Supermix (Bio-Rad) and the CFX96TM Real-Time System (Bio-Rad) were used for amplification with primers. The primer sequences for the promoter region of target genes are listed in Table S1. Raw data were analyzed using the formula ΔCt (MSPI-UD) -ΔCt (HpaII-UD).

*Statistical analysis*

Data are presented as the average ± the standard deviation. The statistical evaluation included the unpaired t-test, Mann-Whitney U test, and two-way ANOVA, all performed using SPSS software version 20 (IBM, Armonk, NY, USA). The application of two-way ANOVA was contingent on the data meeting the criteria for normal distribution and variance homogeneity, and thus, was executed only for groups that conformed to these statistical prerequisites. Statistical significance was set at **p* < 0.05, ***p* < 0.01, and ****p* < 0.001.
